# Effects of the levonorgestrel‐releasing intrauterine device on the immune microenvironment of the human cervix and endometrium

**DOI:** 10.1111/aji.12535

**Published:** 2016-07-12

**Authors:** Uma Shanmugasundaram, Joan F. Hilton, J. William Critchfield, Ruth M. Greenblatt, Linda C. Giudice, Sarah Averbach, Dominika Seidman, Barbara L. Shacklett, Karen Smith‐McCune

**Affiliations:** ^1^ Department of Medical Microbiology and Immunology School of Medicine University of California Davis CA USA; ^2^ Department of Epidemiology and Biostatistics University of California San Francisco CA USA; ^3^ Departments of Clinical Pharmacy and Medicine University of California San Francisco CA USA; ^4^ Department of Obstetrics, Gynecology and Reproductive Sciences University of California San Francisco CA USA; ^5^ Present address: University of North Carolina Chapel Hill NC USA

**Keywords:** chemokine, cytokine, HIV, IUD, progestin, T‐cell

## Abstract

**Problem:**

There is little information regarding the impact of the intrauterine device on immune parameters of the upper female reproductive tract related to risk of HIV acquisition.

**Method of Study:**

We collected cervical and endometrial samples from women using the hormonal intrauterine device to study its effects on endocervical cytokines/chemokine concentrations, phenotypic markers of T cells, responses of endometrial T cells to activation, and alterations of endometrial cellular infiltrates.

**Results:**

Hormonal intrauterine device use was associated with: increased concentrations of inflammatory cytokines/chemokines (endocervix); increased coexpression of CXCR4 and CCR5 (endocervix and endometrium); increased coexpression of CD38 and HLADR (endocervix and endometrium); increased intracellular IL‐10 production after T‐cell stimulation (endometrium); and increased density of T cells, most notably regulatory T cells (endometrium).

**Conclusion:**

Hormonal intrauterine device use resulted in both inflammatory and immunosuppressive alterations. Further research is needed to determine the significance of these changes for HIV risk.

## 1 Introduction

Worldwide, approximately 1 million reproductive‐aged women were diagnosed with HIV in 2013, and among individuals under 25 years old, 60% of new infections were in women.^1^ These findings highlight the importance of understanding the effects of contraceptives on HIV risk. Intrauterine devices (IUDs) are effective long‐acting and reversible intrauterine contraceptives used by approximately 14% of women seeking to prevent pregnancy,^2^, ^3^ and experts have called on the global health community to further increase access to IUDs.^4^ Two types of IUDs are currently available: the non‐hormonal (copper) IUD and hormonal IUDs, which release the synthetic progestin levonorgestrel (LNG‐IUD) locally within the uterine cavity, resulting in relatively low levels of systemic exposure.

Observational studies suggest that systemic exposure to high levels of some progestins may increase risk of HIV acquisition. For example, the time of highest ovarian production of progesterone is 6–10 days after ovulation, which may also be a time of heightened vulnerability for HIV acquisition.^5^ Two recent meta‐analyses report that women using the long‐acting systemic progestin contraceptive, depot‐medroxyprogesterone acetate (DMPA), are at 40–50% greater risk of acquiring HIV than women using no contraceptive or non‐hormonal contraceptives.^6^, ^7^ These findings raise questions about the effects of progestin contraceptives with regard to susceptibility to HIV infection: is risk specific to DMPA, which has unique steroid actions; is risk related to systemic delivery of progestin; or is risk related to long‐term continuous progestin exposure? Thus, it is important to more fully understand the effects of locally released LNG from the LNG‐IUD because IUD contraceptives are highly effective and increasingly used.

The biological basis for a possible association between progestins and HIV risk is not well understood. Some in vitro studies have suggested that endogenous progesterone may lead to increased markers of HIV susceptibility.^5^, ^8^ However, the effect of exogenous progestins on immune function differs by progestin type.^9^ There is little information regarding the possible impact of LNG on innate and adaptive immune responses in the female reproductive tract. Additionally, IUDs are foreign bodies that result in local inflammation in the uterus, which may contribute to both pre‐ and post‐fertilization contraceptive effectiveness.^10^, ^11^, ^12^ Inflammation resulting from the IUD could also result in recruitment of HIV target cells, increasing HIV susceptibility.

Any effect of LNG‐IUDs on susceptibility to HIV infection has not been evaluated rigorously in epidemiologic studies. The limited research that is available has not indicated an association of use of the copper‐containing IUD with an increased risk of HIV infection, but more complete assessment is needed.^13^ The World Health Organization (WHO) medical eligibility criteria for contraceptive use states, “women at high risk of acquiring HIV can generally use LNG‐IUDs,” indicating that “the advantages of using this method generally outweigh the theoretical or proven risks”.^14^ In 2012, the WHO convened a technical panel to address ongoing concerns that hormonal contraceptives might increase the risk of HIV infection. This panel concluded that there is an urgent need for further research in both epidemiology and basic science to evaluate effects of hormonal contraceptives on HIV susceptibility.^15^

The purpose of this study was to determine the effect of a commonly used progestin‐releasing foreign body, the LNG‐IUD, on the properties of mucosal immunity of the upper female reproductive tract. To characterize the unperturbed immune microenvironment, the control group consisted of samples from women not using hormonal or intrauterine contraception that were collected at the time of peak progesterone levels (6–10 days after ovulation). We studied levels of cytokines/chemokines in the endocervical canal, phenotypic markers of endocervical and endometrial T cells, responses of endometrial T cells to activation, and the characteristics of endometrial cellular infiltrates, in order to provide a comprehensive characterization of the effects of LNG‐IUD use on the immune microenvironment of mucosal sites that may support HIV transmission.

## 2 Materials and Methods

### 2.1 Study design

This is a cross‐sectional non‐randomized comparison of women using LNG‐IUD with women using no hormonal contraception. The UCSF Committee on Human Research approved the study protocol, recruiting, and consent materials (approval # 10‐01063).

### 2.2 Recruitment of human volunteers

Healthy women volunteers from San Francisco and the greater Bay Area were recruited via flyers placed in a variety of venues and advertisements in local publications. Participants agreed to refrain from using vaginal products (creams, douches) for at least 10 days prior to study biopsies. Additionally, participants agreed to either abstain from vaginal intercourse or to use non‐lubricated condoms for 72 hr prior to biopsy procedures. Women in the LNG‐IUD groups were required to have had the IUD in place for at least 6 months (Table 1). Women in the control group reported no use of exogenous sex steroids for at least 3 months and a history of at least three normal and consecutive menstrual periods since discontinuation. In addition, if they gave birth in the past year, women in the control group were required to have a history of at least six normal and consecutive menstrual periods. Exclusion criteria included: age <18 or ≥45 years, positive HIV serology, positive urine nucleic acid amplification test for *Neisseria gonorrheae* or *Chlamydia trachomatis*, current or recent pregnancy or breastfeeding, recent gynecological symptoms, recent history of irregular menstrual cycles and/or use of vaginal products, current or frequent genital herpes recurrences, an abnormal cervical cytology in past year, use of other hormonal treatments in past year, use of systemic corticosteroids or immune‐modulating therapies, or daily use of non‐steroidal anti‐inflammatory agents. Also excluded were women unwilling/unable to refrain from vaginal intercourse during the 72 hr before specimen collection. Women received payment to compensate for time and effort required for study participation.

**Table 1:** Baseline demographic and eligibility characteristics by study group

Characteristic	Control (*n*=27)	LNG‐IUD (*n*=19)	*P* values^a^
Age, median (min, max)	34.4 (23.4, 44.3)	26.8 (18.7, 44.4)	.0028
Race, Number (%)			
White/Caucasian	18 (66.7%)	10 (52.6%)	.22
Black/African American	7 (26.0%)	5 (26.3%)	
Native American	0 (0.0%)	1 (5.3%)	
Asian/Pacific Islander	2 (7.7%)	1 (5.3%)	
Other/Mixed	0 (0.0%)	2 (10.5%)	
Current smoking, Number (%)	9 (33.3%)	2 (10.5%)	.19
Proportion ovulated, Number (%)^b^	25 (93)	5 (45)	.003
Months of LNG‐IUD exposure, median (interquartile range)		15.3 (10–25)	

a Data presented as a median was analyzed with a Wilcoxon rank‐sum test, and data presented as *N* (%) was analyzed with the Fisher's exact test.

b Ovulation defined as a serum progesterone of greater than or equal to 2 mg/mL, based on progesterone measurements in all 25 control participants and for 11/19 LNG‐IUD participants.

### 2.3 Clinical study procedures

Women in the control group were instructed to measure their urine for luteinizing hormone (LH) detection using a home detection kit (Clearblue^®^ Ovulation Test DIGITAL, Proctor & Gamble, Cincinnati, OH, USA). Within 7–11 days of urine LH detection (i.e., after ovulation), a study visit occurred for collection of study specimens. Prior to specimen collection, participants were asked whether they had engaged in vaginal intercourse within the prior 72 hr, and if they had, the visit was canceled and the participants were asked to reschedule during the subsequent menstrual cycle. Peripheral blood was collected in EDTA tubes, and serum progesterone was measured to validate the occurrence of ovulation. A speculum was inserted into the vagina, the cervix was visualized, and assessment made for the presence of vaginitis or cervicitis. If vaginal discharge was present, a wet mount was performed. If bacterial vaginosis, candidiasis, or trichomoniasis was diagnosed, the participant was offered treatment and biopsies were not performed. If the exam was normal, the following specimens were collected: endocervical fluid via wick (an ophthalmic sponge [Merocel eye spears, Beaver Visitec International, Waltham, MA, USA] inserted into the canal for 90 s, followed by a second identical collection); endocervical sample using a endocervical cytobrush sample (Cytobrush^®^ Plus Cell collector, CooperSurgical, Trumbull, CT, USA) turned three times; a cervical biopsy (performed at the transformation zone if visible or at the os using a Mini‐Tischler punch biopsy forceps); and an endometrial biopsy using with a 3‐mm cannula (Miltex brand Softflex) inserted through the internal os into the endometrial cavity. For the latter procedure, if insertion of the cannula was difficult, local anesthesia was provided via cervical injection with lidocaine, and then a tenaculum was applied to the ectocervix. A second pass with the curette was made if the amount of tissue was observed to be insufficient after the first pass.

### 2.4 Endocervical wick cytokine/chemokine measurements

**Figure 1:**
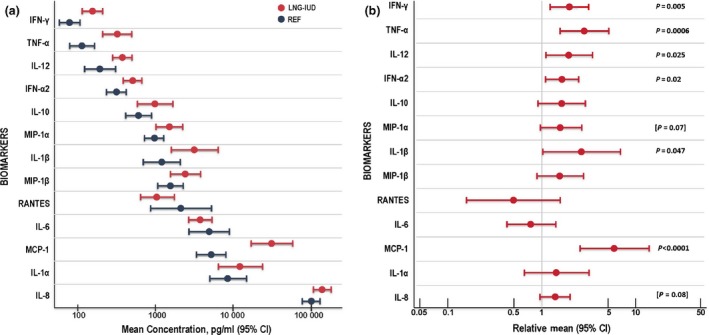
Compares values for 13 cytokines, chemokines, and innate immune factors in endocervical fluids from controls (*n*=24) and LNG‐IUD users (*n*=19). Fluids were collected from participants by insertion of an ophthalmic sponge [Merocel] into the endocervical canal for 90 s; fluids were extracted and analyzed on a Milliplex platform as described in Methods. Panel (a) age‐adjusted mean biomarker concentrations (circles) and 95% confidence intervals (bars) from controls (blue) and LNG‐IUD users (red). Concentration is represented on a log scale in pg/ml, ordered by biomarker concentration in controls from lowest (*y*‐axis top) to highest (*y*‐axis bottom). Panel (b) the mean fold‐change (circle) and 95% confidence intervals (bars) of values from LNG‐IUD users compared to control for each biomarker. Specific *P*‐values are indicated for fold‐change with *P*‐values <.10

Endocervical wick samples were snap‐frozen at the time of collection and stored at −80°C until analysis in bulk (Fig. 1). Wick samples were weighed and then extracted following published techniques into 300 μL ice‐cold extraction buffer (PBS, 0.25 mol/L NaCl + 0.1 mg/mL aprotinin), centrifuged, and extracted a second time in 300 μL of extraction buffer.^16^ Wicks were allowed to air‐dry for 24 hr, then weighed again; the dry wick weight was subtracted from the initial wet weight to determine the net weight (i.e., volume of fluid extracted from each wick). Two wick samples were collected consecutively from each participant, and the two samples were pooled after extraction for each participant for analysis. Samples were assayed on the Milliplex panel (Millipore, Billerica, MA, USA) for the following cytokines: IFNalpha2, IFNgamma, IL‐10, IL‐12p70, IL‐1alpha, IL‐1beta, IL‐6, IL‐8, MCP1, MIP1alpha, MIP1beta, RANTES, and TNFalpha. The plates were read on a Bio‐Plex Suspension Array Reader (Bio‐Rad Laboratories Inc, Hercules, CA, USA). Protein concentrations measured in the assays were multiplied by the dilution factor ([net wick weight + 600] divided by [net wick weight]) to calculate the concentration (pg/mL) of each factor in the wick fluid.

### 2.5 Phenotypic and functional assays of cervical and endometrial T cells

#### 2.5.1 Mononuclear cell isolation

**Figure 2:**
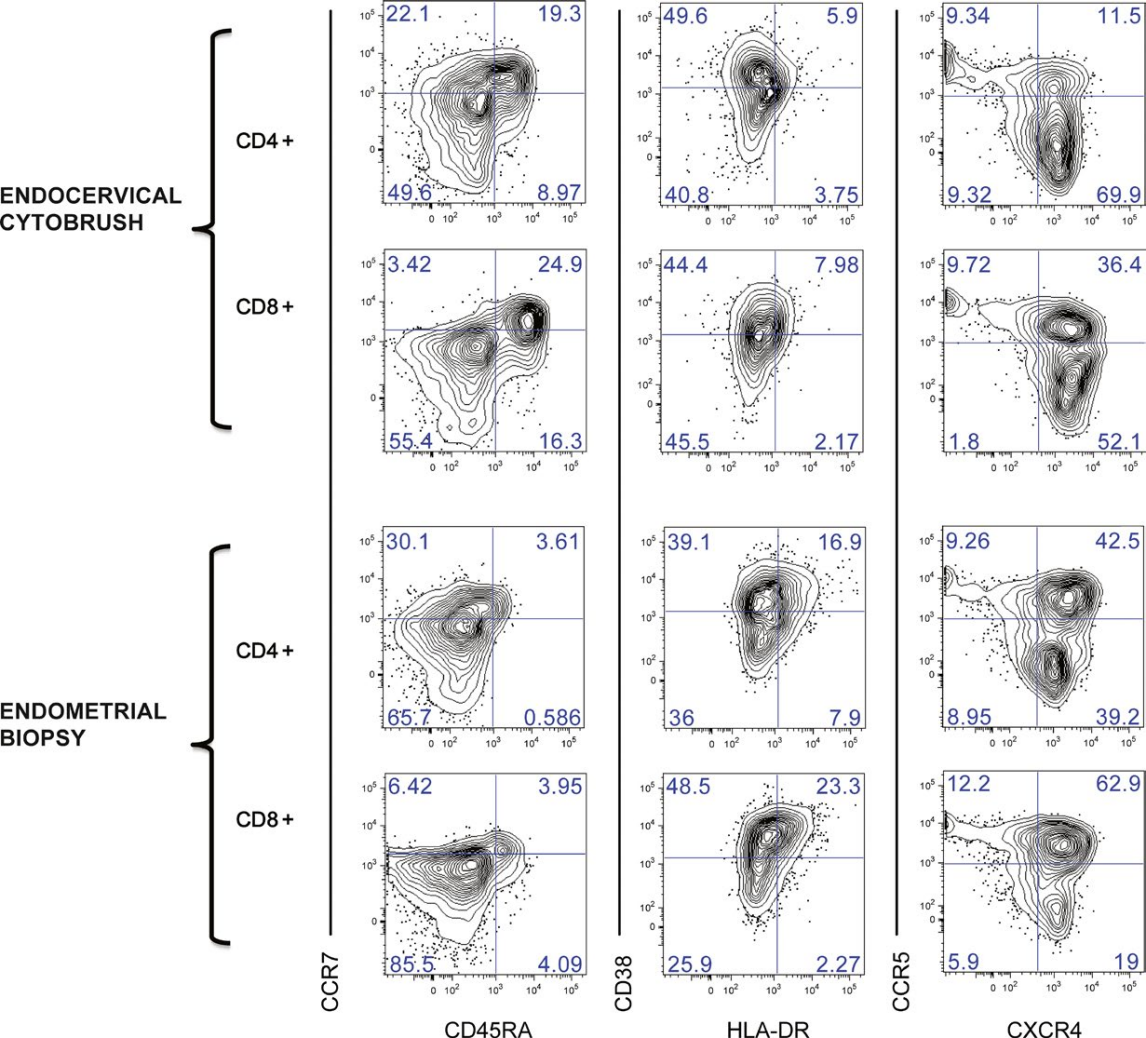
Flow cytometry gating used in the analysis of endocervical and endometrial T‐cell phenotypes. After initial gating of lymphocytes (based on forward vs side scatter) and doublet discrimination, dead cells were excluded by staining with live/dead viable amine; viable cells that were CD3^+^ CD66b^−^ (not shown) were then subdivided into CD4^+^ or CD8^+^ populations. The resulting CD4^+^ or CD8^+^ T cells were then assessed for expression of three pairs of phenotypic markers, as described in the text. Shown from left to right, these were as follows: differentiation markers CCR7 and CD45RA; activation markers CD38 and HLA‐DR; chemokine receptors CCR5 and CXCR4. Quadrant gates were drawn based on fluorescence‐minus‐one (FMO) controls. Numbers in each quadrant indicate percentages of CD4^+^ or CD8^+^ T cells expressing various combinations of markers. Data shown are from a representative participant using LNG‐IUD

**Figure 3:**
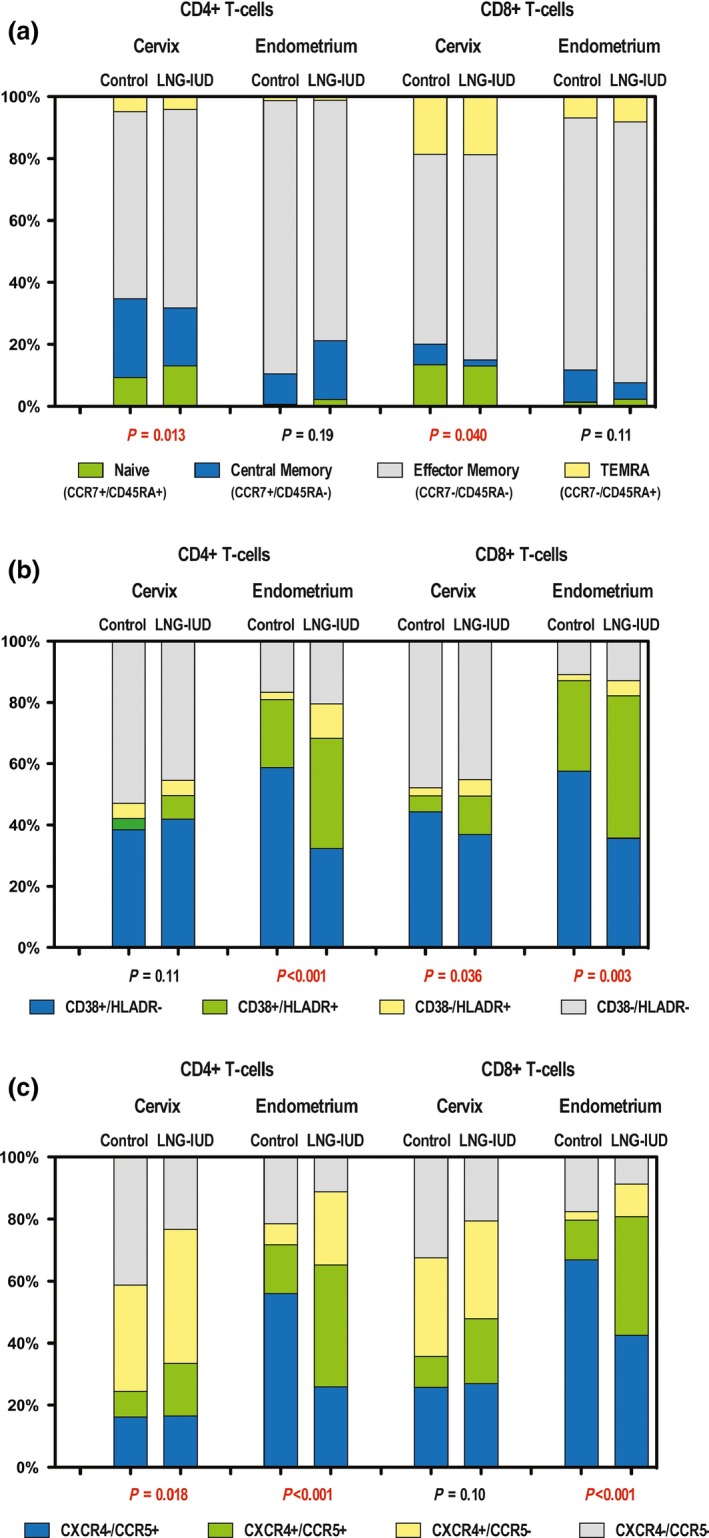
A summary of flow cytometric phenotyping data. Bivariate distributions of three pairs of phenotypes, (a) CCR7/CD45RA, (b) CD38/HLADR, and (c) CXCR4/CCR5, are summarized for CD4^+^ and CD8^+^ T cells from endocervical cytobrush and endometrium, as indicated in the figure headings. The stacked box plots summarize the distribution of each phenotype, analyzed as a function of exposure group (control or LNG‐IUD) in models stratified by tissue and cell type. *P*‐values <.05 are shown in red type. Color coding follows the same pattern in all graphs: +/− blue, −/+ yellow, +/+ green, −/− gray. TEMRA indicates terminally differentiated effector cells expressing CD45RA

**Figure 4:**
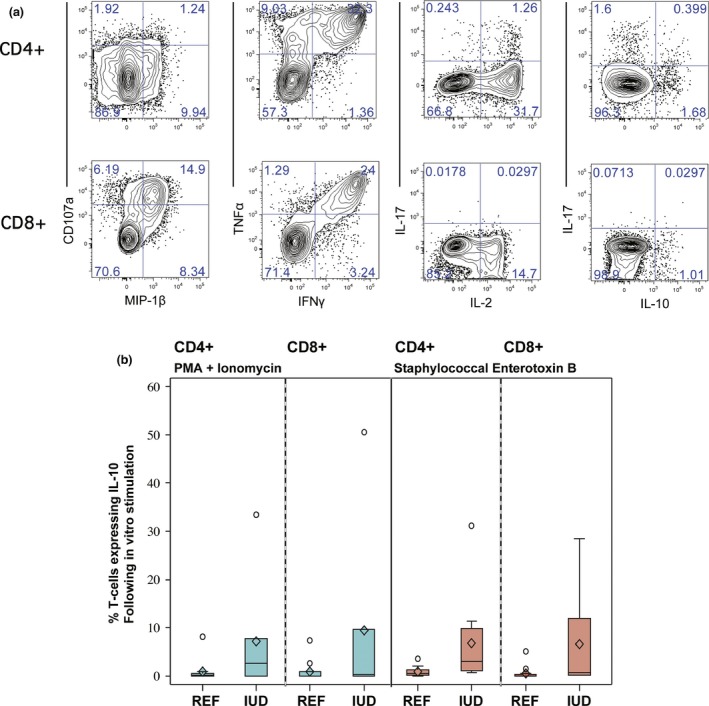
Panel (a) intracellular cytokine production by CD4^+^ and CD8^+^ T cells following polyclonal stimulation. For analysis of endometrial T‐cell responses to stimulation, production of six cytokines (IFN‐γ, IL‐2, IL‐10, IL‐17, MIP‐1β, and TNF‐α) and the granule‐associated membrane protein CD107 were measured by flow cytometry as described in the text. Initial gating was performed to identify lymphocytes and remove doublets (not shown), followed by gating of viable CD3^+^ T cells on subsets expressing either CD4 or CD8, and finally for individual responses as indicated on bivariate plots. Numbers indicate the percentages of CD4^+^ or CD8^+^ T cells in each quadrant. Data shown are from a representative participant using LNG‐IUD with SEB‐stimulated cells. Panel (b) production of IL‐10 by CD4^+^ and CD8^+^ T cells from endometrial tissue of LNG‐IUD users (“IUD”) and control women (“REF”) following stimulation with PMA–ionomycin (blue boxes, left) or SEB (red boxes, right). Box plots illustrate the percent responding cells by subject group, cell type, and stimulus. The length of each box represents the interquartile range (IQR; the distance between the 25th and 75th percentiles), and the interior line represents the median (50th percentile). A symbol (diamond) denotes the mean. “Whiskers” are drawn to the most extreme observations that lie within the *fences*. The *upper fence* is defined as the third quartile plus 1.5 times the interquartile range (IQR), and the *lower fence* is defined as the first quartile minus 1.5 times the interquartile range. Observations outside the fences are identified with small circles

Samples of blood, endocervical brushings, and endometrial biopsies collected at UCSF were transported on ice to UC Davis to arrive within 3–4 hr of collection (Figs 2, 3, 4). Upon arrival, peripheral blood mononuclear cells (PBMC) were isolated by Ficoll–Hypaque (Pfizer, New York, NY, USA) density gradient centrifugation, and washed in PBS. Mononuclear cells from endocervical brushings were isolated by rubbing the brushes together, pipetting several times, passing through a 70‐μm nylon cell strainer (Becton Dickinson, Bedford, MA, USA) and washing in complete medium [RPMI‐1640 supplemented with 15% FBS, penicillin (100 U/mL), streptomycin (100 μg/mL), and glutamine (2 mmol/L)]. Endometrial biopsies were subjected to 2–3 rounds of collagenase type II digestion (0.5 mg/mL; Sigma‐Aldrich, St. Louis, MO, USA), followed by mechanical disruption using an 18‐gauge blunt‐end needle and passage through a 70‐μm nylon cell strainer. The pooled cells were washed in complete medium. Red blood cell lysis was performed as needed on PBMC, endometrium and endocervical cells using ammonium chloride–potassium carbonate–EDTA (ACK).

#### 2.5.2 Monoclonal antibodies for flow cytometry

Fluorochrome‐labeled monoclonal antibodies used for the phenotypic and intracellular staining assay included CD3 (clone UCHT1), CD4 (clone RPA‐T4), CD8 (clone SK1), CCR7 (clone 3D12), CXCR4 (clone12G5), CCR5 (clone 2D7), CD8 (clone RPA‐T8), IFN‐γ (clone B27), TNFalpha (clone MAb11), and MIP‐1beta (clone D21‐1351) from Becton Dickinson Pharmingen (San Diego, CA, USA); CD45RA (2H4) and CD4 (clone T4D11) from Beckman Coulter (Fullerton, CA, USA); CD38 (clone HB7), CD107 (clone H4A3), and IL‐10 (clone JES3‐19F1) from BD Biosciences (San Jose, CA, USA); HLADR (clone TU36) and aqua amine reactive dye from Invitrogen (Carlsbad, CA, USA); CD66b (G10F5) Biolegend (San Diego, CA, USA); and IL‐17 (clone eBio64CAP17) and IL‐2 (clone MQ1‐17H12) from eBioscience (San Diego, CA, USA). Optimum antibody titers were determined empirically for each antibody based on preliminary titration experiments using serial dilutions, which included the manufacturers’ recommended amounts. Fluorescence‐minus‐one (FMO) controls were utilized as needed to establish positive cutoffs.^17^ Flow cytometry data were acquired on an LSRII (BD Immunocytometry Systems, San Jose, CA, USA) equipped with 405, 488, and 643‐nm lasers and utilizing FACSDIVA software (BDIS). Analysis of cytometry data was performed with FlowJo software (TreeStar, Ashland, OR, USA). Results were recorded as the percentage of CD4+ or CD8+ T cells expressing a given surface marker or combination of markers.

#### 2.5.3 Phenotypic and intracellular cytokine staining and flow cytometry

For cell surface phenotyping, endocervical and endometrial cells were stained immediately following sample processing and leukocyte isolation as previously described (Table 2; Fig. 4).^18^ For measurement of intracellular cytokine production, endometrial cells were rested overnight in complete medium prior to performing stimulation assays. The next morning, 2–3 × 10^6^ cells in 200 microliters complete medium were treated with anti‐CD107, monensin (1 μmol/L GolgiStopTM; BD Biosciences), brefeldin A (5 mg/mL, Sigma‐Aldrich, St. Louis, MO, USA), and either staphylococcal enterotoxin B (0.5 μg/mL) or phorbol myristate acetate (PMA, 50 ng/mL) and ionomycin (500 ng/mL). Complete medium containing anti‐CD107, monensin, and brefeldin A served as a negative control. Following a five‐hour incubation, cells were incubated for 5 min in phosphate‐buffered saline (PBS)/2% FCS/0.5 mmol/L EDTA, stained for surface markers and cell viability using aqua amino reactive dye in PBS/2% FCS for 20 min at 4°C, fixed in 4% formaldehyde, then permeabilized using FACS Perm 2 (BD Biosciences). Cells were then washed in PBS/2% fetal calf serum, stained for intracellular cytokines and CD3, washed again, then stored at 4°C in PBS/1% formaldehyde until analysis within 24 hr. The expression of CD107, IL‐2, IL‐10, IL‐17, TNFalpha, IFNgamma, and MIP‐1beta was measured as described previously. Flow cytometry data were acquired and analyzed as described above.

**Table 2:** (A) Endometrial CD4^+^ T‐cell responses to stimulation, ordered by decreasing level among control group, based on samples from 26 participants^a^. (B) Endometrial CD8^+^ T cell responses to stimulation, ordered as in (A), based on samples from 26 participants^b^

Factor	PMA and Ionomycin			Staphylococcal Enterotoxin B (SEB)		
	Control^c^Mean (95% CI)	LNG‐IUD: Control^d^Mean (95% CI)	*P*‐value	Control^c^Mean (95% CI)	LNG‐IUD: Control^d^Mean (95% CI)	*P*‐value
(A)						
TNFalpha	24.5 (14.0–42.8)	0.93 (0.35–2.51)	.89	20.1 (14.6–27.6)	1.36 (0.82–2.24)	.23
IFNgamma	26.4 (15.9–43.8)	0.73 (0.26–2.06)	.55	17.5 (12.9–23.7)	1.09 (0.74–1.62)	.66
IL2	19.4 (11.5–32.7)	0.84 (0.31–2.31)	.74	14.1 (10.5–18.8)	1.3 (0.88–1.98)	.19
MIP1beta	13.1 (7.2–24.0)	0.64 (0.27–1.52)	.31	7.74 (4.80–12.5)	0.91 (0.51–1.60)	.73
CD107	10.5 (5.88–18.8)	***0.23 (0.11–0.48)***	***<.0001***	5.87 (3.55–9.69)	0.66 (0.25–1.72)	.39
IL‐17	1.06 (0.51–2.19)	2.67 (0.89–8.05)	.081	2.17 (1.15–4.12)	1.79 (0.71–4.52)	.21
IL‐10	0.96 (0.26–3.52)	***5.58 (1.11–28.0)***	***.04***	0.96 (0.58–1.59)	***6.05 (2.66–13.8)***	***<.0001***
(B)						
TNFalpha	24.6 (14.0–43.4)	0.43 (0.18–1.02)	.06	20.5 (14.7–28.6)	0.65 (0.39–1.07)	.09
IFNgamma	34.7 (21.7–55.4)	0.83 (0.34–1.99)	.67	22.5 (16.4–31.0)	0.81 (0.54–1.21)	.30
IL2	18.9 (10.5–34.0)	0.65 (0.21–2.05)	.46	10.2 (6.33–16.3)	0.72 (0.40–1.30)	.27
MIP1beta	31.8 (19.1–52.8)	1.08 (0.53–2.20)	.83	18.6 (12.6–27.3)	0.96 (0.57–1.60)	.88
CD107	20.0 (13.7–29.2)	0.60 (0.28–1.31)	.20	18.6 (13.7–25.2)	0.81 (0.50–1.30)	.38
IL17	1.47 (0.46–4.65)	3.3 (0.74–14.7)	.12	1.55 (0.46–5.26)	2.58 (0.56–11.8)	.22
IL10	1.02 (0.33–3.19)	***5.20 (1.13–23.9)***	***.03***	0.55 (0.17–1.81)	***10.7 (2.48–46.4)***	***.002***

CI, Confidence interval.

a PMA and ionomycin results are based on *n*=13 samples for control and *n*=7 for LNG‐IUD; SEB results are based on *n*=16 samples for control and *n*=10 for LNG‐IUD.

b PMA and ionomycin results are based on *n*=12 samples for Control and *n*=7 for LNG‐IUD; SEB results are based on *n*=15 samples for Control and *n*=10 for LNG‐IUD.

c Percent of cells expressing factor among controls.

d Relative percent comparing LNG‐IUD to controls (fold‐change). Results that are statistically significant with *P* value ≤.05 are shown in boldface with italics.

### 2.6 Immunohistochemistry of endometrium

**Figure 5:**
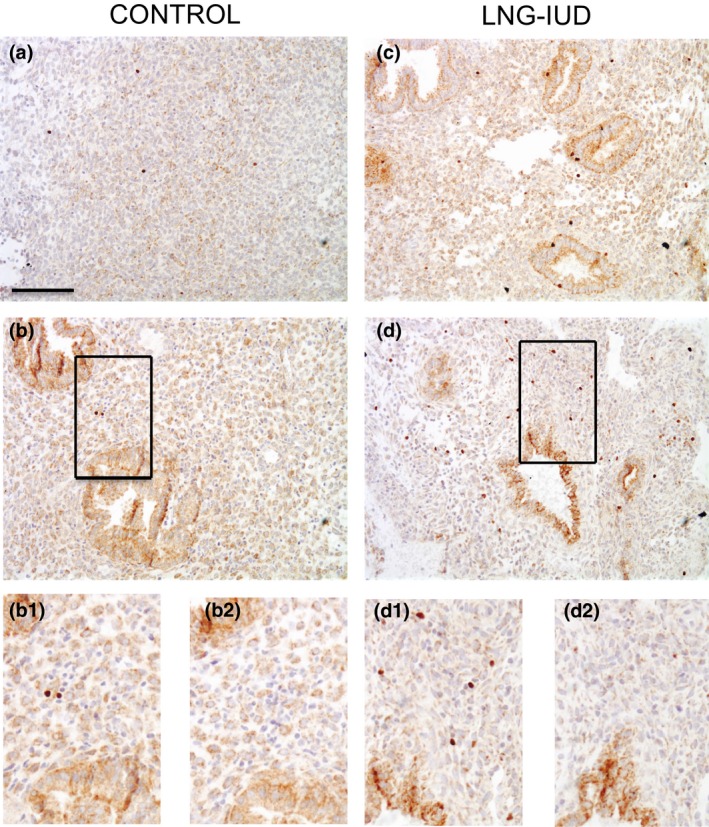
Immunohistochemical detection of regulatory T cells in endometrial biopsies. Five‐micron paraffin sections of formalin‐fixed endometrial biopsies from control participants panels (a and b) and LNG‐IUD users panels (c and d) were incubated with mouse monoclonal anti‐FoxP3 antibodies followed by peroxidase‐conjugated goat anti‐mouse secondary antibodies and diaminobenzidine substrate, as described in Methods. Slides were counterstained with hematoxylin. Dark brown nuclear staining reflects the presence of FoxP3. Enlarged views of rectangular areas outlined in panel (b) and (d) are shown from the same slide panels (b1 and d1) or from a serial section incubated without anti‐FoxP3 antibodies panels (b2 and d2). Bar in panel (a) = 100 μm

At the time of collection, a portion of each endometrial biopsy was fixed in formalin and paraffin embedded (Table 3; Fig. 5). Five‐micron sections were stained with mouse monoclonal antibodies against human CD4 (clone 4B12, 1:10 dilution; ThermoFisher, Rockford, IL, USA); CD8 (clone c8/144B, 1:50 dilution; ThermoFisher); CD56, a marker of NK cells (clone 123c3.d5, 1:100 dilution; ThermoFisher); CD68, a marker of macrophages (clone KP1, 1:100 dilution; DAKO, Carpinteria, CA, USA); and FoxP3, a marker of regulatory T cells (clone 123c3.d5, 1:50 dilution; ThermoFisher) using previously published techniques.^19^, ^20^, ^21^ Peroxidase‐conjugated goat anti‐mouse secondary antibodies (1:200 dilution; Vector Laboratories, Burlingame, CA, USA) and diaminobenzidine substrate (DAKO, kit as directed) were used for detection of antibody binding. Each analysis included an appropriate negative control using mouse IgG1 or IgG2a (DAKO). Photomicrographs of randomly selected fields using the 40× objective of an Olympus BX51 microscope were captured using a CCD camera (Spot Camera; Diagnostic Instruments, Sterling Heights, MI, USA). Positively stained cells were counted on photomicrographs of up 5–10 fields from each slide. As the tissue in the photomicrograph did not always fill the field, the area that contained tissue was outlined and measured on a grid; counts represent the number of stained cells divided by the number of grids included in the image. Tissue areas covering more than 96 grids (of a total possible 382.5 grids) were included in the analysis. To avoid counting projections from a single CD68^+^ cell as multiple cells, CD68 staining had to be associated with a nucleus to be counted as a CD68^+^ cell. To be counted as a FoxP3^+^ cell, the staining had to be nuclear.

**Table 3:** Quantification of cell types by immunohistochemistry in endometrial biopsy specimens, based on samples from 28 participants

Cell type (Antibody)	ControlMean cell density^a^ (95% CI)	LNG‐IUD: Control^b^	
		Fold change (95% CI)	*P*‐value
NK cell (CD56)	71.2 (30.0, 168.7)*n*=10^c^	1.54 (0.63, 3.80)*n*=11^d^	.35
Macrophage (CD68)	55.4 (38.0, 80.6)*n*=12	1.45 (0.93, 2.24)*n*=11	.098
T cell (CD8)	18.8 (11.5, 30.8)*n*=12	***2.63 (1.39, 5.01)**n*=11	***.003***
T cell (CD4)	9.7 (6.0, 15.5)*n*=9	***2.35 (1.21, 4.59)**n*=5	***.012***
Regulatory T cell (FoxP3)	5.54 (3.54, 8.68)*n*=11	***3.52 (2.04, 6.09)**n*=14	***<.0001***

CI, Confidence interval.

a Density is defined as the number of positively stained cells in a standardized unit of area from photographic images of 40× microscopic fields.

b Results that are statistically significant with *P* value ≤.05 are shown in boldface with italics.

c *n* = the number of control samples analyzed.

d *n* = the number of LNG‐IUD samples analyzed.

### 2.7 Statistical methods

#### 2.7.1 Participant characteristics

We summarized and compared characteristics of study participants by exposure group (Table 1). For continuous characteristics (age and duration of current LNG‐IUD exposure), we report median (min, max) and Wilcoxon rank‐sum test *P*‐values. For categorical characteristics (race, smoking status, and ovulation status), we report percentages per group and Fisher's exact test *P*‐values.

#### 2.7.2 Wick analyses

Wick sampling and processing yielded one observation per biomarker per participant (Fig. 1). Preliminary histograms revealed that distributions of protein concentrations (pg/mL) of all biomarkers measured were right‐skewed; consequently, concentrations were analyzed as linear functions of exposure group (LNG‐IUD vs unexposed) and age (centered at 30 years old) using generalized estimating equation (GEE) models, assuming negative binomial distributions and using a log link and robust standard errors. Age‐adjusted group‐specific mean (95% CI) concentrations per biomarker were plotted side‐by‐side, sorted by concentrations in the control group. The corresponding age‐adjusted exposure effects, expressed as a mean (95% CI) relative concentration (fold‐change) among exposed versus unexposed participants, were plotted in the same order. We also report Wald statistic *P*‐values from the GEE analyses.

#### 2.7.3 Flow cytometry

Flow cytometry assays, based one endocervical and one endometrial sample per participant, generated person‐level bivariate distributions of three pairs of phenotypes—CCR7/CD45RA, CD38/HLADR, and CXCR4/CCR5—separately for CD4^+^ and CD8^+^ cell types (Fig. 3). The distribution of each phenotype was analyzed as a function of exposure group in models stratified by tissue and cell type. Using SAS logistic with a glogit link, each person‐level distribution was analyzed as a multinomial outcome, which constrains component percentages (e.g., of CCR7+/CD45RA+, CCR7+/CD45RA−, CCR7−/CD45RA+, and CCR7−/CD45RA−) to sum to 100%. Using SAS genmod with a normal link and accounting for repeated measures per participant, identical mean estimates were obtained by modeling component percentages as a function of exposure group, component level, and their interaction. We report three degree‐of‐freedom interaction‐effect *P*‐values generated by the latter model which are based on empiric standard errors. We present these results via plots of the mean bivariate distributions by cell type, tissue, and arm (Fig. 3).

#### 2.7.4 Immune mediators

Studies of the effects of stimulation of CD4^+^ and CD8^+^ cells from endometrial tissue by PMA–ionomycin or Staphylococcal enterotoxin B (SEB) yielded four observations per immune mediator per participant, expressed as percentages (Table 2; Fig. 4). We used GEE models, assuming negative binomial distributions and using log links and empirical standard errors. Each model expressed log percentage of a phenotype as a linear function of exposure group, stimulant, and their interaction, stratified by cell type. For each biomarker, we report the mean (95% CI) percentage in the control group and the mean (95% CI) exposure effect, expressed as the ratio (fold‐change) among exposed versus unexposed participants (Table 2). Finally, we present box plots of the raw data per exposure group for biomarker IL‐10 to allow comparison of model‐based findings with raw data (Fig. 4b).

#### 2.7.5 Immune cell densities

For each specimen per participant, biomarker cell counts were determined in multiple standardized areas (see Methods for Immunohistochemistry above) (Table 3). Only participants with 5–10 qualifying areas were included in the analysis, generating different sample sizes per marker. Counts of each biomarker were analyzed as above (negative binomial distribution, logarithm link, robust variance estimator) as a function of study group; additionally, an offset used to adjust for the sizes of the areas counted. Results were transformed back to the measurement scale for reporting.

Statistical analyses were conducted using SAS version 9.4. All *P*‐values cited are two‐sided and values less than α = .05 were considered statistically significant.

## 3 Results

### 3.1 Demographics

Nineteen LNG‐IUD users and 27 control women were included in the study (Table 1). LNG‐IUD users were younger than control women (27 years vs 34 years, *P*=.004). The majority of women in both groups identified as white (53% LNG‐IUD users and 67% controls) or black (26% in both groups). LNG‐IUD users were less likely to smoke tobacco than controls (11% vs 33%), but this difference did not reach statistical significance (*P*=.19). The median serum progesterone concentration was lower among LNG‐IUD users compared to controls (0.7 ng/mL vs 7.7 ng/mL, *P*=.03), perhaps due to less frequent ovulation among the LNG‐IUD users (45% vs 93% of controls, *P*=.003). The median duration of LNG‐IUD use was 15.3 months (interquartile range 10–25).

### 3.2 Effects of LNG‐IUD use on chemokines, cytokine, and innate immune factors in the endocervical canal

To determine whether LNG‐IUD altered the immune milieu of the endocervix, we studied concentrations of 13 proteins in endocervical fluid in samples from 19 LNG‐IUD users and 24 control women (Fig. 1). In general, protein concentrations were elevated among IUD users relative to controls, with statistically significant age‐adjusted effects for 6 of 13 endocervical cytokine and chemokine levels. Comparing LNG‐IUD users with control women, significant effects were identified for MCP1 (IUD:control fold‐change 5.97, *P*<.001), TNFalpha (fold‐change 2.83, *P*<.001), IL‐1beta (fold‐change 2.63, *P*=.047), IFNgamma (fold‐change 1.96, *P*=.005), IL‐12 (fold‐change 1.94, *P*=.025), and IFNalpha2 (fold‐change 1.62, *P*=.02). Two other 95% CIs almost exceeded 1.0 (IL‐8 and MIP1alpha). Finally, age was significantly associated with only two protein levels, MIP1beta and TNFalpha, and controlling for age dampened the corresponding IUD effects. These results indicate that LNG‐IUD use is associated with significant increases in soluble inflammatory immune modulators within the endocervical canal.

### 3.3 Effects of LNG‐IUD use on the phenotype distributions of endocervical T cells

Representative flow cytometry gating used in the analysis of endocervical and endometrial T‐cell phenotypes is shown in Fig. 2. To determine whether LNG‐IUD use was associated with changes in immune characteristics of the T cells present in endocervix, the cell surface markers on CD4^+^ and CD8^+^ T‐cell subsets collected via endocervical brushings from 25 controls and 17 IUD users were analyzed by flow cytometry (Fig. 3). In previous studies, we found that when compared with T cells in peripheral blood, a greater proportion of endocervical T cells were activated, effector memory cells,^18^ a pattern we again observed among samples from control women from this study (data not shown). The proportion of CD4^+^ T cells co‐expressing both HIV co‐receptors (CXCR4^+^CCR5^+^) was increased among LNG‐IUD users compared to controls. (Fig. 3c). For both CD4^+^ and CD8^+^ T cells, the proportion expressing markers of the central memory compartment (CCR7^+^CD45RA^−^) was reduced among LNG‐IUD users compared to controls (Fig. 3a). In addition, endocervical CD8^+^ T cells from LNG‐IUD users demonstrated an increase in the proportion of cells expressing the activation marker HLADR (CD38^‐^ HLADR^+^ and CD38^+^HLDR^+^) (Fig. 3b).

### 3.4 Effects of LNG‐IUD use on the phenotype distributions of endometrial T cells

To determine whether LNG‐IUD use was associated with changes in immune characteristics of the T cells present in endometrium, the cell surface markers on CD4^+^ and CD8^+^ (Figs 2 and 3) T cell subsets collected via endometrial biopsies from 16 women per group were analyzed by flow cytometry. In endometrium obtained from control women, >50% of CD4^+^ T cells expressed markers of an effector memory phenotype as well as CD38 and CCR5, as previously reported.^18^ In samples from LNG‐IUD users, the proportions of endometrial CD4^+^ T cells expressing the activation marker HLADR were increased compared with controls (CD38^− ^HLADR^+^ and CD38^+ ^HLADR^+^), whereas the proportions of CD4^+^ T cells expressing CD38 were decreased (CD38^+^ HLADR^−^) (Fig. 3b). The proportions of endometrial CD4^+^ T cells expressing the HIV co‐receptor CXCR4 were significantly increased (CXCR4^+ ^CCR5^−^, CXCR4^+ ^CCR5^+^), whereas those expressing CCR5 without CXCR4 expression were decreased (CXCR4^− ^CCR5^+^) (Fig. 3c).

For CD8^+^ T cells, the proportions of endometrial cells expressing the activation marker HLADR were increased (CD38^−^ HLADR^+^ and CD38^+^ HLADR^+^), whereas the proportion of CD8^+^ T cells expressing CD38 without HLADR was decreased (CD38^+^ HLADR^−^). The proportions of CD8^+^ T cells expressing the HIV co‐receptor CXCR4 were significantly increased (CXCR4^+^ CCR5^−^, CXCR4^+^ CCR5^+^) among LNG‐IUD users, whereas the proportion expressing CCR5 without CXCR4 was decreased (CXCR4^−^ CCR5^+^).

Figure 3 also allows the T‐cell distribution to be compared between the endocervix and the endometrium. As reported previously, T‐cell phenotypes in the two tissues are markedly different despite their anatomic proximity.^18^ By comparing the graphs from control participants for cervix and endometrium, it is apparent that endocervix had significantly greater proportions of naïve, central memory, and terminally differentiated effector CD4+ T cells, as well as higher proportions of naïve and terminally differentiated effector CD8^+^ T cells than T cells recovered from the endometrium (Fig. 3a). However, endometrium had a significantly higher proportion of activated CD4^+^ and CD8^+^ T cells (CD38^+^ HLADR^+^) than endocervix (Fig. 3b). With regard to the distribution of T cells that expressed HIV co‐receptors, the proportions of CD4^+^ and CD8^+^ T cells expressing CCR5 (CXCR4^−^ CCR5^+^ and CXCR4CCR5^+^) were higher in the endometrium (Fig. 3c).

### 3.5 Effects of LNG‐IUD use on the response of endometrial T cells to stimulation

To determine whether the function of endometrial T cells differed between the LNG‐IUD users and control women, we analyzed intracellular expression of soluble immune mediators for CD4^+^ (Table 2A) and CD8^+^ (Table 2B) T cells after stimulation with PMA–ionomycin or SEB. Representative flow cytometry gating used in the analysis of intracellular cytokine production from endometrial T cells is shown in Fig. 4a. In samples from control study participants, after PMA–ionomycin activation, >20% of CD4^+^ T cells expressed TNFalpha and IFNgamma, and ≥20% of CD8^+^ T cells expressed MIP1beta, IFNgamma, TNFalpha, and CD107 (Table 2). In contrast, ≤2% of either CD4^+^ or CD8^+^ T cells expressed IL‐17 or IL‐10 after activation.

Compared to samples from control participants, samples from LNG‐IUD users showed reduced expression of CD107 by CD4^+^ T cells upon PMA–ionomycin activation. In contrast, both CD4^+^ and CD8^+^ T cells from IUD users showed markedly increased production of IL‐10 compared to samples from control participants upon activation by either stimulant, with the fold‐increase ranging from 5.2 to 10.7‐fold (Table 2A and B and Fig. 4b).

### 3.6 Effects of LNG‐IUD use on densities of immune cells in the endometrium

To determine whether the quantity of key immune cell types in endometrium differed between LNG‐IUD users and control women, we performed IHC on endometrial biopsy tissue sections using markers for NK cells (CD56), macrophages (CD68), T cells (CD4^+^ and CD8^+^), and regulatory T cells (FoxP3) (Table 3). In endometrium from control women, NK cells were present at the highest densities, followed by macrophages. CD8^+^ T cells were present at approximately twice the density of CD4^+^ T cells. FoxP3^+^ regulatory T cells were the least common cell type, at a density that could account for approximately half of the population of CD4^+^ T cells in the endometrium.

In endometrial samples obtained from LNG‐IUD users, the densities of NK cells and macrophages were comparable to controls (Table 3). However, the densities of all T‐cell populations were increased in LNG‐IUD users, led by a >threefold increase in regulatory T cells. Examples of IHC staining for FoxP3 in control and LNG‐IUD endometrial biopsies are shown in Fig. 5.

## 4 Discussion

Our results from control participants not using hormonal contraceptives or IUDs provide important normative data about the immune microenvironment of the upper female reproductive tract. In the endocervix, chemokines (IL‐8, MCP1) and proinflammatory cytokines (IL‐1alpha, IL‐6) are present at high concentrations (ng/mL), likely reflecting the role of the cervix in preventing passage of vaginal microflora into the uterus. Our results also highlight important differences between the endocervical and endometrial microenvironments. For example, CD4^+^ T cells expressing activation markers (CD38^+^ HLADR^+^) and the HIV co‐receptor relevant for mucosal HIV transmission (CCR5^+^) are both significantly more abundant in the endometrium than in the endocervix, whereas naïve, central memory, and terminally differentiated effector CD4^+^ T cells are more abundant in the endocervix than the endometrium. These results emphasize the importance of considering each compartment independently in studies related to HIV susceptibility.

Our results indicate that significant differences were identified in the immune microenvironment of the upper female reproductive tract in LNG‐IUD users. In the endocervical canal, LNG‐IUD use was associated with significantly increased concentrations of several inflammatory cytokines (TNFalpha, IL‐1beta, IFNgamma, IL‐12, and IFNalpha2) and a chemokine (MCP1). By virtue of the method of sample collection, we were unable to determine the cellular origin of the cytokines: endocervical cells, infiltrating immune cells, or both. However, as endocervical T cells in samples from LNG‐IUD users were more likely to express activation markers (CD38^+^ HLADR^+^) than samples from controls, some of these proteins were likely derived from local activated immune cells. While the implications of our findings for HIV susceptibility are unknown, the observed higher chemokine concentrations in LNG‐IUD users could result in recruitment of HIV target cells and hence increase HIV susceptibility. Conversely, the observed up‐regulation of inflammatory cytokines could instead indicate a robust local innate immune response that might increase protection against viral infection.

Increased IL‐10 levels in the endocervix have been associated with the presence of sexually transmitted infections,^22^ which are in turn linked to increased HIV acquisition. Our results did not demonstrate a significant increase IL‐10 levels in endocervical fluid of LNG‐IUD users, which is reassuring in terms of HIV risk. However, we did observe a change in HIV target cells in the endocervix; although there was no change in the proportion of CD4^+^/CXCR4^−^ CCR5^+^ T cells in LNG‐IUD users, there was an increase in CD4^+^ T cells expressing both CXCR4^+^ and CCR5^+^. Given that transmitted HIV virions are predominantly CCR5 tropic, and that the columnar epithelium of the endocervix is considered a favored site for HIV transmission, our finding of an increased proportion of HIV target cells at that site may have important implications for HIV susceptibility in LNG‐IUD users.

Our results indicate that use of the LNG‐IUD was associated with increased expression on endometrial T cells of the HIV co‐receptor CXCR4 and decreased expression of CCR5. These results are similar to those reported by a study of the effects of LNG‐IUD and copper IUD on endometrial T cells 2 months after IUD insertion, which showed decreased expression of CCR5 in the endometrium of LNG‐IUD users.^23^ Participants of our study had been exposed to LNG‐IUDs for a much longer period of time; all had used the LNG‐IUD for at least 6 months, with median use time of 15 months, indicating that the reduction in CCR5^+^ endometrial T cells observed after 2 months of device use was sustained over longer periods of use. As in the endocervix, we did observe an increase in the endometrium of CD4^+^ T cells expressing both CXCR4^+^ and CCR5^+^. Endometrial macrophages have also been shown to be a potentially important target for HIV infection^24^; hence, it is reassuring that our results also did not demonstrate an increased density of macrophages in the endometrium of LNG‐IUD users.

The presence of a foreign body such as the IUD in the uterus may result in an inflammatory response and constitute a primary mechanism for preventing pregnancy.^10^, ^11^, ^12^ Indeed, our results indicate that a higher proportion of CD4^+^ and CD8^+^ T cells co‐expressing the activation markers CD38 and HLADR are present in the endometrium of LNG‐IUD users compared to controls, suggesting a response to local perturbations either from the IUD itself or the local release of LNG. In addition, transcriptional profiling of endometrial samples from participants of this study (previously published) revealed that the endometrial transcriptome in LNG‐IUD users is characterized by marked up‐regulation of pathways regulated by inflammatory mediators TNFalpha, IL‐1beta, and NFkappaB.^25^ Regulatory T cells are often recruited in response to and in order to control local effects of chronic inflammation. Indeed, our immunohistochemical analysis showed significantly increased numbers of regulatory T cells in the endometrium in LNG‐IUD users compared to controls. In addition, we found that activation of endometrial T cells was associated with increased production of the immunosuppressive cytokine IL‐10 in LNG‐IUD users. These results indicate that the endometrium of LNG‐IUD users is poised more for a Th2 (immunosuppressive) than a Th1 (cytotoxic) response compared to controls, perhaps indicating that the endometrium employs compensatory mechanisms to regulate chronic inflammation induced by the presence of a foreign body. Furthermore, while the role of IL‐10 in HIV susceptibility and disease progression is likely complex, polymorphisms associated with decreased IL‐10 production have been associated with increased susceptibility to HIV infection.^26^ Therefore, the elevated levels of IL‐10 after immune activation in LNG‐IUD users in this study may indicate an HIV‐protective mechanism.

The strengths of our study are the wide range of immune parameters that were measured, and the collection of samples from comparison participants timed to a narrow window of the ovulatory cycle, thus minimizing the effects of ovarian sex steroid variations on our findings. In addition, by enrolling women who had chronic exposure to the LNG‐IUD, we were able to characterize the impact of this IUD in a timeframe that better mirrors actual use than post‐insertion observations. This study is limited by relatively small numbers of individuals and samples analyzed, the fact that absence of recent sexual activity was documented by self‐report only, and that the LNG‐IUD group had a lower percentage of women who had ovulated compared to controls. Ultrasound studies have demonstrated that ovulation occurs in a subset of women using the LNG‐IUD.^27^ We assume that the local release of LNG would dominate any effects of progesterone released cyclically in the women who ovulated, but were unable to determine whether the release of progesterone from ovulation might have contributed to our findings, or perhaps masked a larger effect from the LNG‐IUD had we controlled for this variable. Additionally, we cannot infer the effects of LNG‐IUD directly as we did not study women prior to and after device insertion. We were unable to determine whether our results reflect the presence of a foreign body versus the local release of LNG in the endometrium. Comparison of effects of the copper (non‐hormonal) IUD to those of LNG‐IUD will help distinguish between these possibilities.

Our results indicate LNG‐IUD use resulted in both inflammatory and immunosuppressive changes (the latter being perhaps compensatory in nature) in the local immune microenvironment of the endocervix and endometrium. Further research is needed to determine the effects of these changes on HIV infection of target cells from LNG‐IUD users, and on overall risk of HIV acquisition.
